# Primary signet ring cell carcinoma with tubular adenoma of the rectum: A case report and a review of the literature

**DOI:** 10.1097/MD.0000000000020985

**Published:** 2020-06-26

**Authors:** Yong-Ping Yang, Ling-Yun Yu, Jian Shi, Jian-Nan Li, Xin-Yu Wang, Tong-Jun Liu

**Affiliations:** a Department of General Surgery, The Second Hospital of Jilin University; b Department of Ear Nose and Throat Surgery, The First Hospital of Jilin University, Changchun, China.

**Keywords:** primary signet ring cell carcinoma with adenoma, radical resection, rectum

## Abstract

**Rationale:**

Among the various forms of colorectal carcinomas, primary signet ring cell carcinoma (SRCC) of rectum is infrequent. Primary SRCC with adenoma is even rarer. Due to its low morbidity and lack of obvious manifestations at early stages, it is difficult to make an early diagnosis and perform surgical intervention in time. Herein, we reported a case of primary SRCC with tubular adenoma of rectum and also performed a review of the literature of such cases, in hopes of expanding the general understanding regarding such cases.

**Patient concerns:**

A 61-year-old male patient presented with rectal bleeding for 1 week.

**Diagnoses:**

A neoplasm could be palpated through a rectal examination, with a size of 4.0 cm by 3.0 cm, at a distance of 5 cm from the anal edge. Magnetic resonance imaging examination and colonoscopies were performed to confirm the finding, and 4 tissue specimens were obtained for histopathologic biopsy. The result of biopsy was high-grade intraepithelial neoplasia with an adenoma component.

**Interventions:**

Surgical resection was performed, and histopathologic and immunohistochemical staining examination of the resection confirmed the diagnosis of SRCC with tubular adenoma.

**Outcomes:**

The patient was discharged from hospital 12 days postsurgery, without any complications. Further chemotherapy and supportive treatments were suggested to him and will be followed at a local hospital.

**Lessons:**

Primary rectal SRCC has a rather low morbidity. Furthermore, a rectal SRCC with adenoma which was presenting in this case is even more rare. Besides lack of clinical characters, delay of diagnosis and treatment frequently occur. So far, a surgical procedure has still been one of the most effective treatments. Considering of metastasis and the poor prognosis, early diagnosis, in-time radical resection, and a comprehensive followed treatment are recommended for a higher 5-year overall survival.

## 1 Introduction

Colorectal carcinoma (CRC) is one of the major causes of cancer mortality worldwide.^[1]^ Colorectal signet ring cell carcinoma (SRCC) is a rare subtype of CRC, manifesting at a low rate of about than 1%.^[2]^ The term of SRCC is a descriptive term, denoted by the histologic observation of cell nuclei being pushed to the periphery due to excess intracytoplasmic mucin.^[3–5]^ SRCC was firstly reported in 1951 by Laufman and Saphir.^[6]^ The majority of SRCC cases are found in stomach, although some cases have also been reported in breast, lung, bladder, pancreas, and colon-rectum.

Since the clinical manifestations typically appear late, colorectal SRCC is frequently detected at an advanced stage. Furthermore, SRCC tends to be more aggressive than carcinomas of other histologic types, leading to poor prognosis for colorectal SRCC cases.^[7]^ As such, improvements that allow for more timely diagnosis and earlier surgical intervention may significantly improve the 5-year survival rates and overall outcomes.

**Figure 1:**
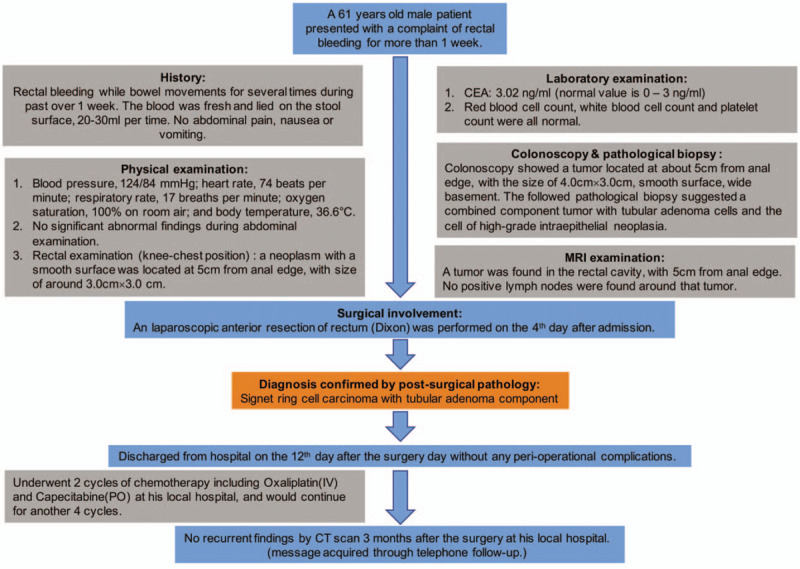
Timeline organizing the main events of the case.

However, because of lack of obvious clinical characteristics of rectal SRCC, and poor awareness by the doctors, misdiagnosis of such tumors may occur, and some patients may consequently miss the optimum opportunities for intervention. Here, we reported a case of rectal SRCC with rectal adenoma components and its clinical characteristics, and hope to raise appreciation for the possibility of such rarer cases during clinical diagnosis (Fig. 1).

## 2 Case presentation

A written informed consent statement was obtained from the patient following approval from the Institutional Ethics Committee of the Second Hospital of Jilin University.

A 61-year-old male patient presented with a complaint of rectal bleeding for more than 1 week, with no abdominal pain or any changes of bowel movement. He denied having any history of recent fever, chills, nausea, vomiting, or weight loss. The patient had a history of smoking for around 25 years, of 20 cigarettes per day. He denied any history of drinking, or other concomitant diseases. His father died from heart disease, and his mother died from stomach carcinoma.

A physical examination showed us that his overall condition was relatively normal: blood pressure, 124/84 mm Hg; heart rate, 74 beats per minute; respiratory rate, 17 breaths per minute; oxygen saturation, 100% on room air; and body temperature, 36.6°C. Abdominal examination also had no significant abnormal findings. However, rectal examination (knee-chest position) found that a neoplasm with a smooth surface was located at 5 cm from anal edge. Its size was around 3.0 cm × 3.0 cm. The root of this neoplasm could be moved slightly by palpation. No dark red blood or pus remains were found on the glove when the rectal examination was finished.

**Figure 2:**
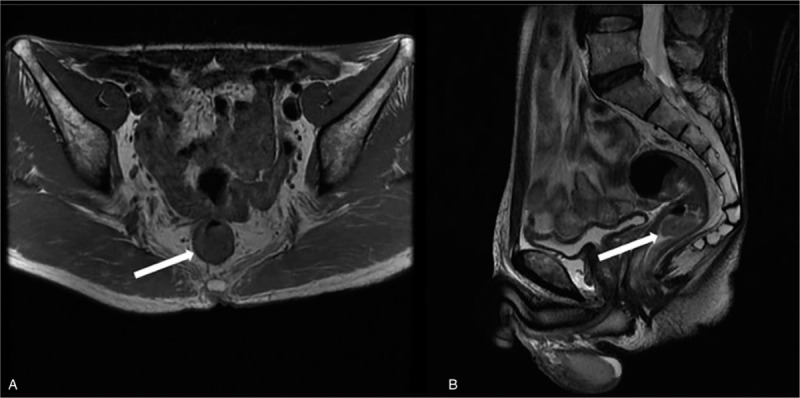
The manifestation of magnetic resonance imaging. (A) T1 manifestation in cross section view. The tumor size was 23 mm × 10 mm × 10 mm (with white arrow). (B) T2 manifestation in sagittal view. The distance from distal edge of tumor to anal edge was around 55 mm (tumor with white arrow).

Routine laboratory examinations showed that the patient had relatively normal complete blood counts, with white blood cell count, 5.7 × 10^9^ cells/L (normal range is 3.5–9.5 × 10^9^ cells/L); red blood cell count, 4.53 × 10^12^ cells/L (normal range is 4.30–5.80 × 10^12^ cells/L); platelet count, 208 × 10^9^ cells/L (normal range is 125–350 × 10^9^ cells/L). However, the patient did show slightly increased carcinoembryonic antigen (CEA) levels. A colonoscopy confirmed the presence of a tumor at about 5 cm from the anal edge, with a size of 4.0 cm × 3.0 cm, smooth surface, wide basement, and surrounded by congestive mucosa. Four tissue specimens were obtained from this tumor for pathologic biopsy. The result of this biopsy revealed that tubular adenoma cells were found, together with cells of high-grade intraepithelial neoplasia. A consequent nuclear magnetic resonance imaging (MRI) examination confirmed that tumor was in the rectal cavity, with the location as same as that described during colonoscopy, with no swollen lymph nodes being found around the rectum (Fig. 2).

Based on the family history, the results of laboratory examinations, the coloscopy report, the result of pathologic biopsy, and the MRI report, a diagnosis of rectal carcinoma was made. The patient and his family were consulted about the indication and risks of the following surgery, whereupon they provided written consent to undergo a laparoscopic anterior resection of rectum (Dixon) for the rectal carcinoma. During the surgery, we found that tumor in the rectum at a distance of about 6 cm distance from anal edge, with no obvious evidence of invasion of the tumor toward the surrounding tissue. Laparoscopic total mesenteric excision was performed, and several enlarged suspected lymph nodes were found during surgery. No complications occurred during or postsurgery. That patient was discharged from hospital 12 days postsurgery.

**Figure 3:**
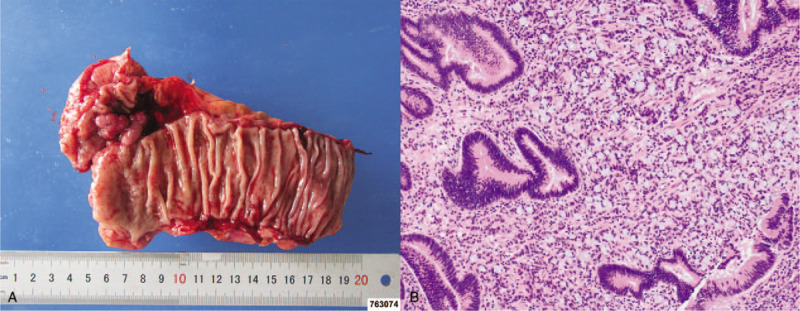
Histopathologic examination for postsurgical specimen. (A) The resected rectal lumen removed from the patient's body. (B) Histologically, the resected specimen showed a signet ring cell carcinoma manifestation, with the invasion into the submucosal layer. Meanwhile, a component of tubular adenoma was also found surrounding (100× by hematoxylin-eosin staining).

Subsequent pathologic examination of the resected surgical specimen supported the diagnosis of rectal SRCC with tubular adenoma. From the serial sectioning of the surgical specimen, the histology assessment showed scattered foci of signet ring carcinoma cells invading the lamina propria nearby the adenomatous lesion (including both low- and high-grade intraepithelial neoplasia) (Fig. 3). Meanwhile, 2 in 11 perimesenteric lymph nodes were found to have infiltrating carcinoma cells. However, no evidence was found to support the possibility of a vascular infiltration. As a result, the TNM staging was T1N1bM0. An immunohistochemical examination was followed. The outcomes were as follows: P53 (40% positive), CDX2 (positive), Ki67 (50% positive), CD34 (vasculature positive), PMS2 (positive), MLH1 (positive), MLH6 (positive), MLH2 (mild positive), VE1 (negative), CgA (negative), Syn (negative), CD56 (negative), Villin (positive), CK (AE1/AE3) (positive), TTF-1 (negative), CK7 (negative), CK20 (positive), SATB2 (positive), D2-40 (vasculature positive), and CD31 (vasculature positive).

After his discharged from hospital, a 6-month follow-up was performed by telephone. He was then asked to perform a blood examination to monitor levels of CEA and CA-199. Both CEA and CA-199 fell in the normal range postsurgery. Neither a computed tomography scan performed 3 months postsurgery or an MRI performed 6 months postsurgery found evidence of potential recurrence. At the same time, the patient also underwent 6 cycles of XELOX chemotherapy. The patient did not report experiencing any serious adverse effects, such as neutropenia, neurotoxicity, or bone marrow suppression.

## 3 Discussion

The SRCC is a rather rare subtype of CRC. According to previously published literature, tits incidence ranges from 0.1% to 0.9% among all forms of CRC.^[8]^ Due to the low incidence, SRCC has only been evaluated sporadically in a limited numbers of cases. However, SRCC is almost always reported to be found at an advanced stage and with a poor prognosis, as compared with the more common forms of common adenocarcinoma (CAC) and mucinous adenocarcinoma (MAC).^[4,5,9,10]^

### 3.1 The origin of SRCC

There has been considerable debate about the histologic origin of SRCC for decades. Four main theories have been proposed: CAC, due to the finding of transition areas between typical CAC and SRCC in many cases^[11]^; adenoma, typically reported as a combination of adenoma cells and signet ring cells, just as this case^[11–15]^; atypical epithelium, which is associated with p53-positive intraepithelial signet ring cells^[11]^; and a combination of these lesions.

A distinct pattern of Kras mutations and lower Kras mutation frequency has been reported for colorectal SRCC cases as compared with that of CAC.^[16]^ Meanwhile, an A:T transversion at the 3rd base position of K-ras codon 61 has also been reported. Additional biomarkers, such as Reg IV and claudin-18, have been reported to be more highly expressed in SRCC as compared to CAC, and both markers have been previously implicated to be involved in gastric cancer.^[17]^ HATH1, MUC2, and SOX2, identified as the key genes involved in controlling mucin secretion in the gastrointestinal tract, have also been reported to be more highly expressed in SRCC, consistent with histologic findings of excess mucin buildup.^[17–20]^

### 3.2 SRCC as an independent prognostic factor

In previous studies, patients’ age, gender, tumor TNM stage, tumor location, and treatments have been confirmed as prognostic factors for various forms of CRC, including SRCC. However, SRCC subtyping has also been shown to be a significant as an independent prognostic factor associating with 5 years overall survival.^[4,5,9,10,21–24]^ To confirm this significance, we further analyzing the TNM staging data for cases of SRCC, CAC, and MAC from our hospital over the past 2 years (from September 2017 to September 2019, shown in Table 1). Overall, SRCC cases tended to have a more advanced TNM staging compared with CAC and MAC, with the ratio of T3+T4 vs total T in SRCC being higher than others. The same phenomena could be seen with N stage (N1+N2 vs total N) and M stage (M1 vs total M) of SRCC. Additionally, the immunohistochemical characteristics of the rectal SRCC cases in our group were tabulated in Table 2. According to these analyses, a higher proportion of Ki67+ cells were found, with similar trends for CDX2, PMS2, and MLH1, while being P53 negative, suggesting that the tumors were likely to more rapidly proliferate. As a consequence, we believe that previous indications that SRCC cases are likely to be more aggressive and advanced staged are indeed accurate.

**Table 1:**
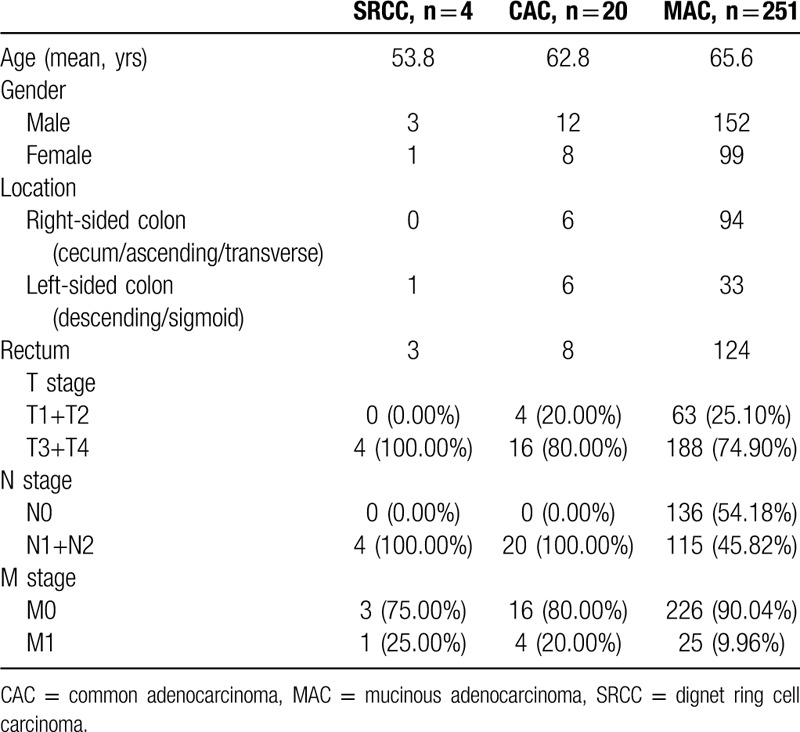
Clinical data of SRCC, CAC, and MAC of colon and rectum in our group from September 2017 to September 2019.

**Table 2:**
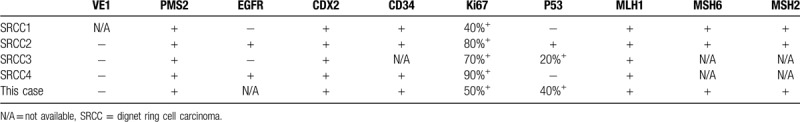
Immunohistochemical characteristics of the rectal SRCC cases in our group.

To further confirm our findings, we subsequently performed a meta-analysis of the relevant literature as summarized in Table 3. These studies also corroborated this trend. Additionally, Inamura et al proved that SRCC component within CRC always come with a prospect of a higher recurrent mortality, <50% of SRCC component associated with cancer-specific mortality hazard ratio of 1.40 and >50% of SRCC component associated with that ratio of 4.53.^[7]^

**Table 3:**
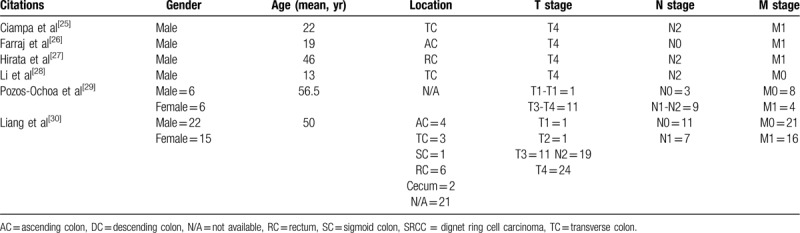
Main clinical-pathologic characters of colorectal SRCC cases reported in literatures for recent 2 years from June 2017 to September 2019.

### 3.3 Local invasion, lymph and distant metastases at relative early stage

The SRCC has a trend of a local invasion, with lymph and distal metastases often being found.^[9,21,31,32]^ Colorectal SRCC also has a higher possibility of peritoneal metastases, and more frequently invades via the lymphatic route, compared with CAC and MAC. A poor prognosis and lower survival rate are strongly indicated when the evidence of peritoneal metastases is confirmed.^[33]^

In this present case, an adenoma component was found in SRCC tissue. Normally, this phenomenon would suggest that the lesion was found at an early stage of tumor growth. However, adenoma with SRCC could only indicate histopathologic origin but cannot serve as suggestive evidence without confirmation of local invasion or metastasis. Unfortunately, lymph nodes invasion was found during the surgery, which was confirmed consequently by the report of pathologic examination of the surgical specimen. As such, our report further indicates that rectal SRCC may invade and/or metastasize at early stage, even if a component of adenoma is found in tumor tissue.

### 3.4 Approaches to the treatment of colorectal SRCC

Normally, as with the other subtypes of CRC, a timely surgical resection of colorectal SRCC has been considered to be the most effective treatment with the highest promise of overall survival.^[34,35]^ Additionally, since SRCC may sometimes develop into peritoneal metastasis, cytoreductive surgery and hyperthermic intraperitoneal chemotherapy is recommended on patients with select manifestations of colorectal SRCC.^[36]^ Hugen et al argued that although SRCC subtyping was a risk factor for poorer prognoses, cases of SRCC would still benefit from adjuvant chemotherapy similar to other forms of CRC.^[37]^ At the same time, Fu et al held the viewpoint that SRCC patients would benefit little from the resection of primary and metastatic lesions after they had reviewed 3568 patients with CRC with or without signet ring cell by multivariate analysis.^[38]^ However, only a small number of colorectal SRCC cases have been evaluated thus far (only 94 in the cohort reported on by Fu et al, with a 11.5% higher invalid operation rate), and larger studies may be necessary to clarify the true utility of surgical resection for such patients.

## 4 Conclusion

Due to the low morbidity and lack of defining clinical characteristics, rectal SRCC is usually diagnosed at an advanced stage. However, SRCC may be considered as a possibility in cases of hematochezia of unknown cause. Since SRCC is typically more aggressive and has higher risks of metastasis, timely detection and surgical intervention, and comprehensive follow-up treatments are recommended to ensure better patient outcomes.

## Acknowledgments

The authors appreciate the contributions of all the surgeons, coworkers, and friends involved in this study and thank the editors and reviewers for their help with this manuscript.

## Author contributions

Yong-Ping Yang and Tong-Jun Liu conceived of this study, designed it. Yong-Ping Yang and Ling-Yun Yu drafted the manuscript. Yong-Ping Yang, Ling-Yun Yu, Jian Shi, and Jian-Nan Li participated in design of this study, acquired the data and analysis of data. Xin-Yu Wang acquired the data. Yong-Ping Yang and Tong-Jun Liu participated in manuscript preparation and critical revision. All the authors read and approved the manuscript.
